# Morphological covariates of the ontogenetic shift from nauplii to copepodite prey in larval fish

**DOI:** 10.1111/jfb.70014

**Published:** 2025-03-12

**Authors:** Pierre Pepin

**Affiliations:** ^1^ Northwest Atlantic Fisheries Centre Fisheries and Oceans Canada St. John's Newfoundland Island Canada; ^2^ Present address: Three Dog House 1023 Indian Meal Line Portugal Cove St. Philip's Newfoundland Canada

**Keywords:** body size, empirical model, foraging success, prey selectivity, prey–predator interactions

## Abstract

Larval fish are active planktonic predators, with many species feeding initially on copepod nauplii and gradually shifting their selection to copepodites. This study evaluated whether it is possible to develop a general widely applicable empirical model to describe the transition from feeding on copepod nauplii to copepodites in relation to body length, maxilla length and eye diameter. The study also evaluated whether the switch to copepodites is linked to what prey are in the stomach or which copepodite species are replacing nauplii in the diet was also considered. The high degree of covariation among the three morphometric variables makes it difficult to establish statistical differences among predictive models. However, the highest overall fit to a logistic model and accuracy in the transition from nauplii to copepodites are achieved when eye diameter is used as a predictive variable. There are also fewer significant differences in the residuals among fish taxa in the case of eye diameter relative to the other morphometric variables. Fish taxa that shift their diet from copepod nauplii to prey on cyclopoid copepodites demonstrate a slower ontogenetic transition away from nauplii than taxa that shift to calanoid copepodites. Eye diameter may be a stronger predictor of the shift in diet because it contributes to most of the processes that make up the foraging sequence of larval fish prey–predator interactions.

## 1 INTRODUCTION

Many fish species produce large numbers of small eggs from which precocious larvae emerge, still dependent on parentally derived energy reserves that are essential to survival for the first few days of life (Hjort, 1914; Houde, 2008; Hunter, 1981). The reproductive strategy of fish leaves larvae highly vulnerable to starvation unless they can effectively find, capture and consume suitable prey prior to the exhaustion of their yolk. Over a period of days to weeks, individuals undergo substantial changes in body size, morphology, swimming ability and physiology, along with improved cognitive development and capacity to respond to changes in their predator–prey environment (Deary et al., 2017; Herbing & Gallager, 2000; Leis, 2007; Osse, 1990; Ronnestad et al., 2013). The larval phase, from hatch to metamorphosis, consists of a period when the mass of individual larvae may increase 100‐ to 1000‐fold. Such substantive changes are often associated with an important ontogenetic shift in diet composition (Heath & Lough, 2007; Llopiz, 2013; Pepin, 2023).

Most larval fish are active planktonic predators and demonstrate a high degree of flexibility in which prey they consume, and the pattern of prey selection is highly dependent on the latitude and nature of the zooplankton community in which larvae forage (Llopiz, 2013). Gut analyses reviewed by Llopiz (2013) revealed that copepods are the most common prey type among most fish species and ecosystems, with Calanoida and Cyclopoida the dominant orders, although dependence on Appendicularia, bivalve larvae and Cladocera is also widespread. Copepod nauplii make up a high proportion of the diet of larval fish across all latitudes but their importance declines as ontogenetic development progresses (Last, 1978a, 1978b; Morote et al., 2008, 2010; Pepin, 2023). The length‐dependent larval fish ontogenetic shift from copepod nauplii to copepodites is highly species specific (Peck et al., 2012; Pepin, 2024), with no clear understanding or theory of which factors may be governing the switch between copepod developmental stages, although prey and predator body size are likely important factors.

Understanding what factors result in changes in reliance on nauplii versus copepodites can provide an indication of what larval developmental state may involve a critical transition that can affect survival potential. There is no underlying theory that would guide predictions of prey selection or the transition from feeding on copepod nauplii to feeding on copepodites, but there are a number of factors or principles to consider in evaluating available observations (Miller et al., 1988, 1992). Three morphological features can provide insights into general patterns in prey selection (Peck et al., 2012): (1) body length, linked to swimming speed (Leis, 2007; Peck et al., 2012) and prey capture ability (Heath, 1993; Morley & Batty, 1996); (2) gape (dependent on maxilla or jaw length), which defines the maximum width of prey that can be ingested (Munk & Kiørboe, 1985; Munk, 1992, 1995; Pepin & Penney, 1997; Shirota, 1970); and (3) eye diameter, linked to retinal development, which determines visual acuity (Blaxter, 1968, 1986; Poling & Fuiman, 1997). Body form, reflecting differences in musculature and body depth, may also factor into larval attack capacity, although Williams et al. (1996) found limited differentiation in the rapid acceleration escape response of five species of larval fish with differing morphologies. This study evaluated whether it is possible to establish general principles, applicable across fish species, describing the transition from feeding on copepod nauplii to feeding on copepodites based on morphological descriptors of body dimensions in larval fish. Whether the switch to copepodites is linked to what prey are in the stomach or which prey types are replacing nauplii in the diet was also considered.

The data for this study are from a sub‐Arctic coastal ecosystem (~1000 km^2^) in the northwest Atlantic. Previous analyses of these data identified strong patterns of selection for various prey types and sizes (Pepin & Penney, 1997), and that feeding success was linked to prey diversity along with morphological and behavioural differences among individuals (Pepin, 2023, 2024), based on comparisons among 11 species of larval fish. Pepin (2023) found that maxilla and body lengths, within and among fish taxa, have a dominant and positive influence on larval feeding success and that differences in larval swimming capacity and/or prey perception/capture ability are likely important factors contributing to that success. Importantly, Pepin (2023) noted that as larvae increase the number of prey in their stomachs, the mean width (i.e. size and body mass) of prey decreases, which is not consistent with optimal foraging theory. Pepin (2024) concluded that prey selection was achieved through increases in apparent prey perception and/or larval responsiveness to dominant prey types (i.e. nauplii and copepodites) and that foraging success increased modestly with larger eye diameter and mouth gape, possibly owing to different capacities to perceive and attack prey. It is noteworthy that none of the studies of this ecosystem were able to identify consistent patterns in the feeding ecology related to taxonomic proximity for the 11 species included in the analyses. Copepods dominated the diet in all larval fish taxa, with Calanoida and Cyclopoida nauplii being most frequent, which provides data and patterns comparable to findings from earlier reviews (Llopiz, 2013; Nunn et al., 2012; Peck et al., 2012). The analyses presented below focus entirely on the change in the proportion of nauplii and copepodites, with the contribution of non‐copepod prey types excluded from the assessment.

## 2 METHODS

Plankton was sampled fortnightly from late May until late September in 1985 and 1986 at three locations on the eastern shore of Conception Bay (47°45′N, 53°00′W), Newfoundland, Canada (see Pepin & Penney (1997) for details about the region and the location of sampling sites) to provide a comprehensive assessment of the seasonal variation in larval feeding patterns (Pepin, 2023, 2024). Ichthyoplankton were collected using a 0.75 m ring net, 3.5 m in length, fitted with 165 μm nitex and a General Oceanics flow meter (to assess volume filtered), and towed at a depth of 5–10 m at a speed of 0.3–0.5 m s^−1^ for 10 min. Samples were preserved in 4% buffered formaldehyde.

All fish larvae were sorted and identified to the lowest taxonomic level possible, and individuals were measured for standard length to the nearest millimetre using an ocular micrometre, with subsamples of larvae of 11 dominant species of fish taken for stomach analysis (Table 1). For each individual, maxilla length (or jaw, where the maxilla had not formed) was measured to the nearest 10 μm and the digestive tract was removed and teased apart using fine dissecting needles. Stomach contents were identified to the lowest taxonomic and life stage possible. For prey with definitive stages, such as copepods, the width of five individuals from individual zooplankton samples was measured to the nearest 5 μm, which revealed consistency in prey dimensions throughout the sampling period. All calanoid copepod nauplii could not be identified reliably to species level, with the exception of *Calanus finmarchicus* and *Temora longicornis*. The remainder of the calanoid copepodite community in coastal Newfoundland is dominated numerically by *Pseudocalanus* spp. (Pepin et al., 2011; Pepin, unpublished data; Maillet et al., 2022) so it is highly likely that the majority of unidentified calanoid nauplii reflected that of the copepodite community. Cyclopoid nauplii and copepodites were identified as *Oithona similis*. Details of stage width and occurrence for the four dominant nauplii taxa are listed in Table S1.

**TABLE 1:** Larval fish sample characteristics.

Species (family)	Common name	Number of specimens	Number with prey	Range in length (mm)	Average Fulton *K* (SD)	Total prey enumerated
*Clupea harengus* (Clupeidae)	Atlantic herring	146	135	6–16	0.20 (0.066)	482
*Mallotus villosus* (Osmeridae)	Capelin	187	111	4–20	0.18 (0.048)	937
*Gadus morhua* (Gadidae)	Atlantic cod	206	189	3–20	1.26 (0.57)	4989
*Glyptocephalus cynoglossus* (Pleuronectidae)	Witch flounder	272	246	4–18	0.35 (0.18)	2437
*Hippoglossoides platessoides* (Pleuronectidae)	American plaice	176	138	3–18	0.76 (0.36)	1156
*Myzopsetta ferruginea* (Pleuronectidae)	Yellowtail flounder	190	175	2–12	1.09 (0.63)	1052
*Pseudopleuronectes americanus* (Pleuronectidae)	Winter flounder	202	164	2–7	1.01 (0.32)	890
*Tautogolabrus adspersus* (Labridae)	Cunner	184	165	2–9	1.13 (0.46)	1067
*Ulvaria subbifurcata* (Stichaeidae)	Radiated shanny	154	147	6–14	0.79 (0.22)	2206
*Stichaeus punctatus* (Stichaeidae)	Arctic shanny	92	89	6–15	0.72 (0.19)	1136
*Liparis* spp. (Liparidae)	Snailfish	171	151	3–15	1.76 (0.27)	857

*Note*: The order of fish species is based on the phylogenetic classifications used by Pepin (2023). Average and standard deviation (SD) of Fulton's *K* ($K=1000\times \left(\text{weight}/{\text{length}}^{3}\right)$, where weight is in mg dry and length is in mm (Ricker, 1975)) were estimated from the species‐specific length–weight relationships from Pepin (1995) and based on the number of 1 mm length intervals of larvae from this study.

The proportion of nauplii relative to the total number of copepod prey in the diet (nauplii + copepodites) was averaged among individuals for each estimated value of each morphological metric for each fish taxa. The average maxilla length of fish was estimated based on direct measurements for each 1 mm length interval, and eye diameter was estimated based on the species‐specific length‐dependent linear relationships reported by Pepin (2024) (Table S2). Uncertainty in maxilla length and eye diameter was estimated from the standard deviation of the residuals of their relationships with length (Table S2). The standard deviation in measurements of body length was estimated as 0.14 mm based on the repeated measurements from Pepin et al. (1998).

### 2.1 Analyses

Three analyses were performed to determine whether a general model of transition from nauplii to copepodites was feasible and informative. First, a four‐parameter logistic curve was fitted to the pattern of change in the proportion of nauplii in the diet (y) in relation to morphological metrics x (body length, maxilla length, eye diameter), a quantitative representation of the ontogenetic shift in diet, such that:

(1) $$y=\min+\left[\left(\max-\min\right)/\left(1+{\left(x/\mathrm{EC}50\right)}^{-b}\right)\right]$$

where min and max are the lower and upper asymptotes of the relationship, EC50 describes the point at which nauplii make up 50% of the copepod prey in the diet and b is the rate of change in diet composition in relation to x, the metric of body morphology. Parameters were estimated using nonlinear least squares regression. Fits to the logistic relationships were compared based on adjusted *r* ^2^, standard errors of the estimates, *F* ratios and change in Akaike information criterion (ΔAIC) (Burnham & Anderson, 2002). Model fit among morphometric variables was contrasted using *F* ratios. Species‐specific correlation of the residuals of the model for each morphological metric were evaluated against each of the other metrics, as well as length‐specific estimates of Fulton's condition index ($K=1000\times \left(W/L^{3}\right)$, where *W* is wight in mg dry weight and *L* is length in mm (modified from Ricker (1975), see Nash et al. (2006): a greater *K* indicates increased body depth (i.e. mass) at a given length) for each fish species based on the allometric relationships from Pepin (1995). Fulton's *K* was treated as a macroscopic indicator of larval body morphology (e.g. form, depth, musculature) among fish taxa across the range in length for each species, with the standard deviation among length classes providing a measure of changes in body form during development for each fish species. The correlation analysis aimed to evaluate how well the logistic model represents the pattern of change in nauplii contribution for each fish species. Negative correlations indicate a more rapid shift to copepodites than the model predicts and vice versa for positive correlations.

Second, a general linear model analysis of variance (ANOVA) was applied to the residuals of the logistic model to assess whether there are any significant differences among fish taxa (as a classification variable) for the logistic model outcomes fitted to each of the three morphological metrics. Overall model results were contrasted using *F* ratios and comparisons among the estimated least square means for each fish taxa were performed to determine which species differed from others based on *Bonferroni* corrections for multiple assessments (*α* = 0.05/55). This correction provides a conservative assessment of differences among fish taxa that reduces the likelihood of type I error (false positive) and sets high expectations to identify significant differences among fish taxa. However, *Bonferroni* corrections reduce the potential for type II error (false negative) that can result from large samples sizes based on categorical averages, as in this study, that can yield high levels of precision in model estimates of the mean that may exceed that possible given uncertainty in the measurement of morphological variables.

Finally, a principal components analysis of the residuals for the best fitting logistic model was performed to assess how they were related to the mean and variability in nauplii width in the stomach, the proportion of calanoid nauplii, larval length and the relative proportions of the copepodites in the diet (*O. similis*, *Pseudocalanus* spp., *T. longicornis* and *C. finmarchicus*), based on normalized data. This analysis assesses how the characteristics of prey in the stomachs of larvae, and the prey types to which selection is shifting, affect the ontogenetic shift from nauplii to copepodites. The rationale is based on Pepin's (2024) findings that larvae with more prey in their gut demonstrated unbalanced differences in prey selection relative to individuals with fewer prey in their gut. Pepin (2024) found an important shift toward feeding more extensively on *Oithona similis* and *Temora longicornis* copepodites and a relative shift away from *Pseudocalanus* copepodites. Pepin (2023) found that increased weight‐corrected differences in relative gut fullness were associated with increased ingestion of a greater number of smaller prey.

### 2.2 Larval fish morphology

Larval length varies from 2 to 20 mm, with differences between smallest and largest larva within taxa ranging from 5 to 17 mm. Overall ranges among taxa for maxilla length and eye diameter are 0.0952.07 and 0.116–1.45 mm, respectively. Both maxilla length and eye diameter are highly significantly positively correlated with body length (Table S2 and Figure S1). Overall, there is greater change in maxilla length than eye diameter with increasing body length. The relative average rate of increase in eye diameter relative to maxilla length is 0.63, ranging from 0.35 to 0.99 among fish taxa. However, there is greater variability among taxa in the slope of the length‐dependent relationship for eye diameter (3.52‐fold) relative to maxilla length (2.24‐fold). The relationships between eye diameter and maxilla length demonstrate less differentiation among taxa (i.e. more overlap) than in the relationships of each metric with body length (Figure S1). This pattern is also apparent in the relationships of the three morphological variables with estimated body weight (Figure S2), with the highest degree of differentiation occurring between body length and weight. There is also greater variability among taxa in the standard deviation of the residuals of the length‐dependent relationships for eye diameter (3.35‐fold) than for maxilla length (1.98‐fold), although the average absolute standard deviation of residual variability among individuals is greater for maxilla length (0.065 mm) than eye diameter (0.054 mm) (Table S2). The uncertainty (standard deviation of length‐dependent relationships relative to the range of each morphometric variable) in these morphological variables based on the variability among individuals (Table S2) is ~2%–5%, with uncertainty in body length ~0.5%–5% based on Pepin et al. (1998). The maximum width of nauplii in the stomachs is less than the gape of the mouth in all fish taxa (Figures S3 and S4), and the distribution and median prey width relative to gape of the mouth are similar for both nauplii and copepodites (Figure S4).

## 3 RESULTS

**FIGURE 1:**
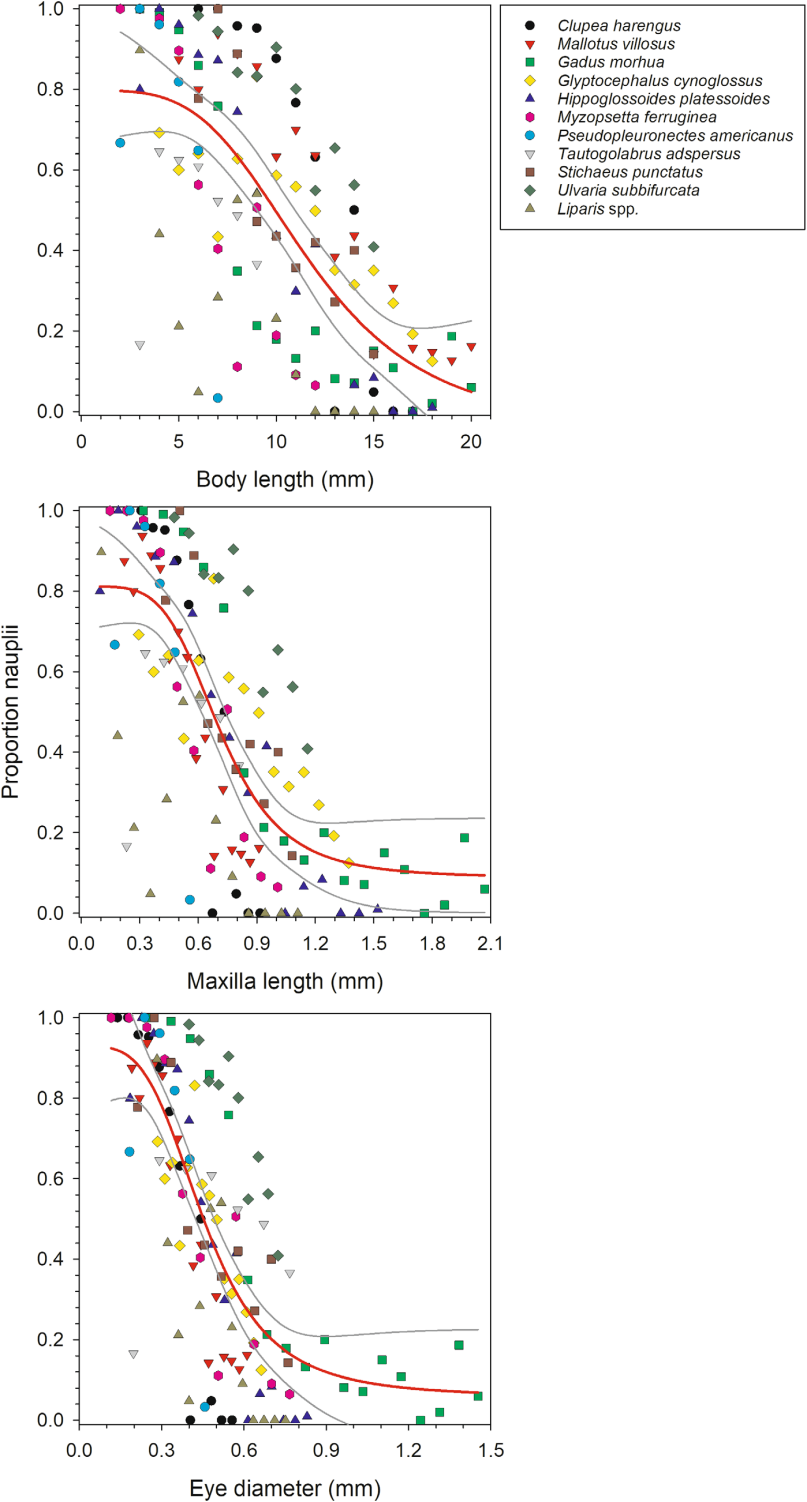
Proportion of nauplii, relative to all copepods, in the diet of larval fish in relation to body length (top panel), maxilla length (middle panel) and eye diameter (bottom panel), with symbols representing each fish taxa. The red line represents the logistic curve estimated for each morphometric variable. Grey lines represent the 95% confidence intervals of the model relationships.

**FIGURE 2:**
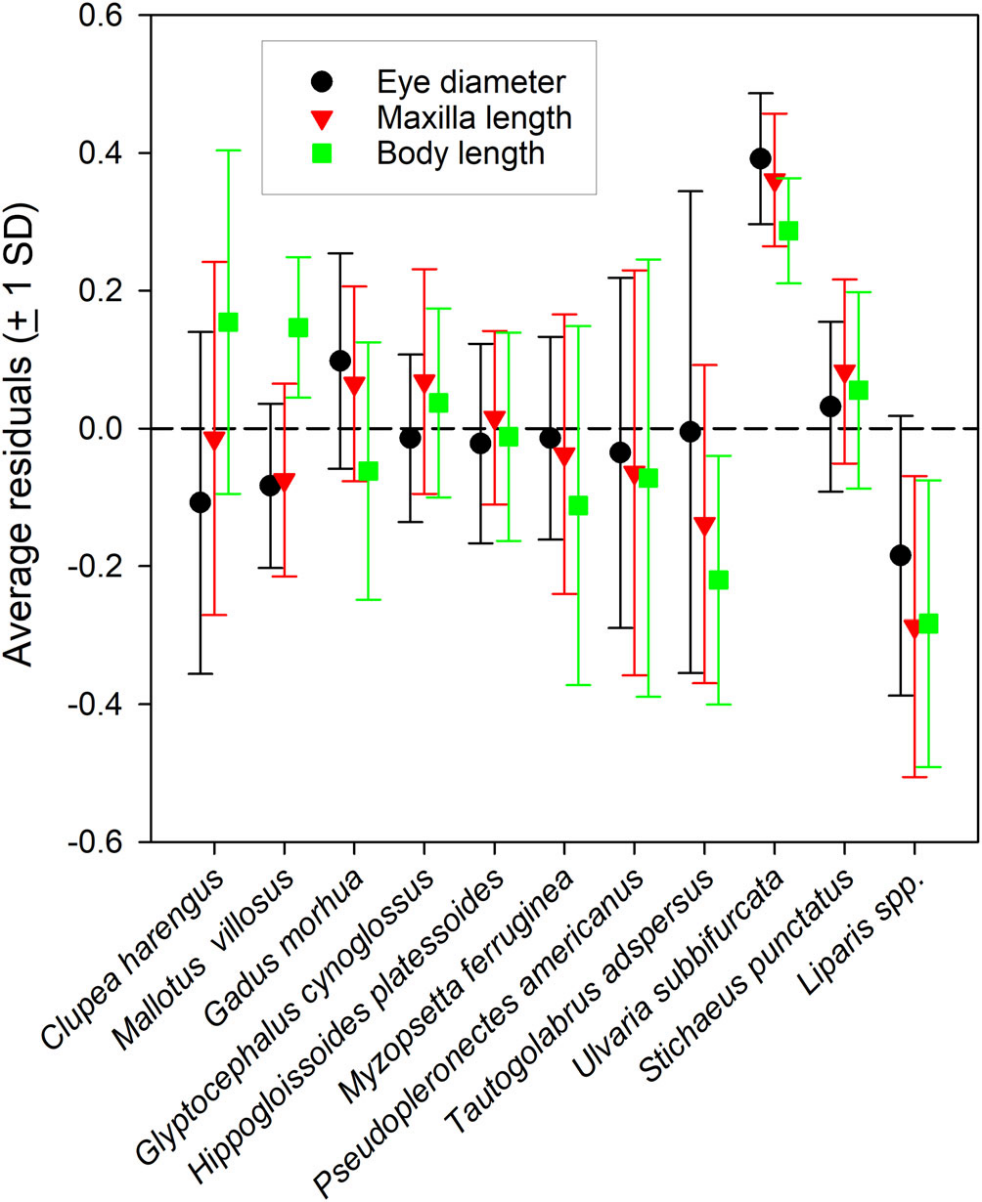
Mean and standard deviation of the residuals of the logistic model for each of the morphometric variables for each fish taxa. The dashed line represents the zero reference.

A four‐parameter logistic curve, fit to the pattern of change in the proportion of nauplii in the diet in relation to the three morphometric variables, describes well the ontogenetic shift in the proportion of nauplii in the diet. The logistic model provides a highly significant fit (*p* < 0.001) for all morphometric variables (Figure 1 and Table 2). The adjusted *r* ^2^ ranges from 0.50 to 0.58. Model fit is strongest for eye diameter, followed by maxilla length and body length. The *F* ratios of the difference in the fit of the models of the relationships for eye diameter and maxilla length, and eye diameter and body length are 1.07 and 1.14, respectively, and not significantly different among morphometric variables (*p* > 0.1). However, the ΔAIC relative to the best fitting model for eye diameter reveals differences of 11.6 (*p* < 0.01) for the comparison with maxilla length and 20.9 (*p* < 0.001) for the comparison with body length. None of the estimates of min are significantly different from 0 (*p* > 0.2) but estimates of max for the logistic models for maxilla and body length are significantly less than 1 (*t* = 3.62 and 3.23, respectively, *p* < 0.001) whereas the estimate of max for the logistic model for eye diameter is not significantly different from 1 (*t* = 0.87, *p* > 0.1). Estimates of EC50 represent 65%, 36% and 34% of the range in body length, maxilla length and eye diameter, respectively. However, the overall uncertainty in EC50 is ±19.5% of the range in length among fish species, whereas it is ~±10.5% of the range of both maxilla length and eye diameter, indicative of a more accurate definition of the transition from feeding on nauplii versus copepodites for the latter two metrics (Figure 2). Relative uncertainty in estimates of EC50 is more than 2–10 times greater than relative uncertainty in measurements of morphological features. Residuals from the logistic fit to eye diameter are the only ones to pass the Shapiro–Wilk test for normality (*p* > 0.05) and the test for constant variance (*p* > 0.5). Despite the limited significant differences among overall model fits, weight of evidence indicates that eye diameter appears to be a better predictor of the dietary transition from copepod nauplii to copepodites.

**TABLE 2:** Parameter estimates and their standard error (SE), for the logistic models of the proportion of copepod nauplii in the diet, relative to all copepods, in relation to body length, maxilla length and eye diameter, along with adjusted *r* ^2^, standard error of the model estimate and *F* value for each model.

Metrics and parameters	Body length		Maxilla length		Eye diameter	
	Estimate	SE	Estimate	SE	Estimate	SE
Min	−0.052	0.219	0.088	0.0773	0.058	0.0886
Max	0.797	0.063	0.812	0.052	0.929	0.0813
EC50	11.7	1.79	0.715	0.0535	0.456	0.0355
*b*	−3.76	1.60	−4.49	1.40	−3.78	1.11
Adjusted *r* ^2^	0.50		0.53		0.58	
Estimated SE	0.237		0.229		0.219	
*F* value	45.8		52.3		61.0	

Correlation analysis among the residuals aims to describe the pattern of covariation in the misfit to the logistic models among morphological features of the larvae. The residuals from the three logistic models demonstrate a high degree of overlap with the expected value of 0 among fish taxa (Figure 2), indicative of generally similar degrees of uncertainty. Residuals from the three models are highly significantly correlated (*r*_body‐maxilla_ = 0.82, *r*_body‐eye_ = 0.71, *r*_maxilla‐eye_ = 0.93, *p* < 0.001). Overall, residuals from the logistic model for eye diameter are not significantly correlated with Fulton's *K* (*r* = 0.10, *p* > 0.2), but are significantly negatively correlated with body length (*r* = −0.19, *p* < 0.05), suggesting they are independent of body mass. Fulton's *K* is significantly negatively correlated with the residuals for maxilla length (*r* = −0.22, *p* < 0.05) and body length (*r* = −0.53, *p* < 0.001) models, in contrast to eye diameter.

Analysis of variance of the residuals was applied to assess the degree of differentiation among fish taxa. The analyses, based on a comparison of species‐specific average residuals, revealed highly significant differences among fish taxa for all three morphometric metrics (*p* < 0.001) (Table 3). The weakest effect of fish taxa is for eye diameter and it is greatest for body length. Two‐sample *F* tests for comparison of variances among models revealed no significant differences (*p* > 0.1) among the different model results for all three morphometric metrics (*F* = 1.09 for eye diameter and maxilla length, *F* = 1.17 for eye diameter and body length). However, pairwise comparisons among taxa, based on *Bonferroni* corrections for multiple assessments, revealed substantially different patterns in which taxa are significantly different from one another (Figure S5). For eye diameter, radiated shanny (*Ulvaria subbifurcata*) is different from all other taxa, clearly evident in Figure 2, and Atlantic cod (*Gadus morhua*) is different from snailfish (*Liparis* spp.). However, differentiation among taxa widened for the residuals of the logistic model for maxilla length, with additional differences between snailfish and several other taxa (Figure S5). Differentiation increased to several other pairs of taxa for the residuals of the logistic model for body length (Figure S5) but did not appear to be strongly affected by taxonomic proximity. Relaxing the *Bonferroni* corrections for multiple assessments would have yielded 19, 24 and 29 significant differences among taxa for eye diameter, maxilla and body length, respectively. A lower analytical threshold resulted in increases in significant differences for two clupeid‐like larvae, capelin (*Mallotus villosus*) and Atlantic herring (*Clupea harengus*), with most other taxa, particularly in the case of comparisons with maxilla and body length, as well as four more significant differences for cunner (*Tautogolabrus adspersus*), two with maxilla length and two with body length. In the case of *Liparis* spp., seven, nine and eight of the comparisons with other taxa are significant at *p* < 0.05, for eye diameter, maxilla and body length, respectively, relative to two, seven and five identified based on *Bonferroni* corrections. Despite the lack of significant differences among model fits there were fewer significant differences among taxa in the case of the residuals of the logistic model for eye diameter than for maxilla or body length.

**TABLE 3:** Generalized linear model analysis of variance results used to assess differences among taxa in the residuals from the logistic models used to describe variation in the proportion of nauplii in the diet relative to each of the morphological metrics.

Metric	Source	DF	SS	MS	*F* value	Significance
Body length	Taxa	10	3.13	0.313	9.22	<0.001
	Error	123	4.17	0.034		
	Total	133	7.30			
	Adjusted *r* ^2^	0.43	RMSE	0.184		
Maxilla length	Taxa	10	2.86	0.286	8.92	<0.001
	Error	123	3.95	0.032		
	Total	133	6.81			
	Adjusted *r* ^2^	0.42	RMSE	0.179		
Eye diameter	Taxa	10	2.43	0.243	7.84	<0.001
	Error	123	3.81	0.031		
	Total	133	6.24			
	Adjusted *r* ^2^	0.39	RMSE	0.176		

Abbreviations: DF, degrees of freedom; MS, mean square; RMSE, root mean square error; SS, sum of squares.

Significant correlations of body length with the residuals from the three logistic models were highly variable but mostly negative for Atlantic herring, capelin, Atlantic cod and American plaice (*Hippoglossoides platessoides*), and positive for witch flounder (*Glyptocephalus cynoglossus*) and cunner (Figure S5). Significant correlations of Fulton's *K* with residuals from the three logistic models were equally variable, with negative associations for Atlantic herring, witch flounder and American plaice, and positive associations for capelin, Atlantic cod and cunner (Figures S5). Negative correlations indicate a more rapid shift to copepodites than the logistic models predicted in relation to length or Fulton's *K* for individual taxa, and vice versa for positive correlations.

**FIGURE 3:**
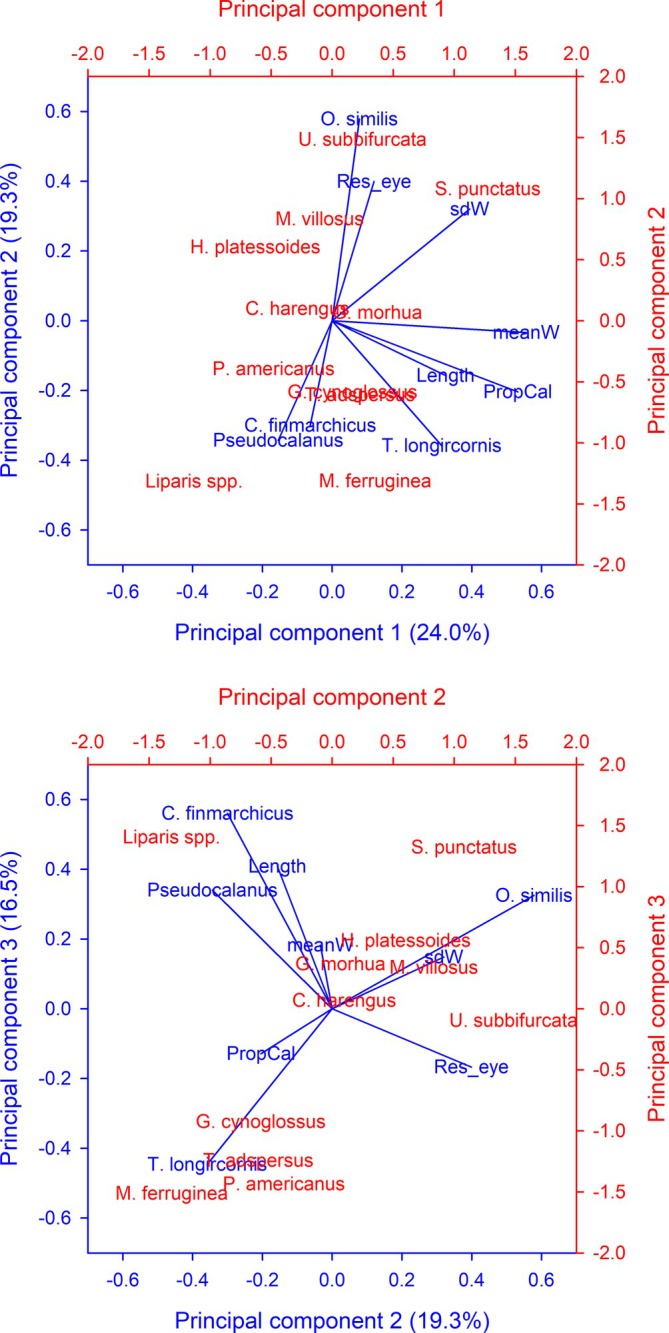
Biplots of the principal components loadings (in blue, bottom and left axes) for mean (meanW) and standard deviation (sdW) of nauplii width, proportion of calanoid nauplii (PropCal), larval length (Length), the residuals of the logistic model for eye diameter (Res_eye) and the proportions of copepodites of *Oithona similis*, *Pseudocalanus* spp., *Calanus finmarchicus* and *Temora longicornis*, along with average principal component loadings for the 11 fish taxa included in the analysis (in red, top and right axes; symbols represent abbreviated genus and species names).

Principal components analysis was performed to assess how the characteristics of prey in the stomachs of larvae, and the prey types to which selection shifts, affect the ontogenetic shift from nauplii to copepodites. The three significant principal component axes (*λ* > 1) explained 24%, 19.3% and 16.5% of the variance in the relationship between diet characteristics and the residuals of the logistic model fit to eye diameter (Figure 3). Larval length, proportion of calanoid nauplii in the diet, mean and standard deviation in nauplii width, and the proportion of *T. longicornis* copepodites loaded positively on the first principal component. The residuals of the logistic model loaded positively on the second principal component, along with the proportion of *O. similis* copepodites, while the proportion of *Pseudocalanus* spp., *C. finmarchicus* and *T. longicornis* copepodites loaded negatively, indicating differences in the shift to different copepodite species. Importantly, the trends related to the size of nauplii in the stomachs and the proportion of calanoid nauplii on the first principal component were orthogonal to the loading of the model residuals on the second principal component, indicating that the trends associated with these factors are generally independent of the shift to different copepodites species. The second and third principal components highlighted the separation in prey selection based on the copepodite contribution to the diet from different prey taxa to the diet of the different fish taxa. These two axes also serve to emphasize the separation between snailfish and the two species of shanny, as well as the grouping of three flatfish species (witch, winter [*Pseudopleuronectes americanus*] and yellowtail [*Myzopsetta ferruginea*] flounders) with cunner. The groupings of Stichaeidae/Liparidae and that of the three Pleuronectidae demonstrated some degree of influence from taxonomic proximity on the ontogenetic shift from nauplii to copepodites. Overall, the patterns in the principal components analysis highlight that prey types replacing copepod nauplii in the diet have a strong influence on deviations from the general logistic model.

## 4 DISCUSSION

Strong covariation among body length, maxilla length and eye diameter resulted in limited statistical differentiation among fits to the logistic model to the three metrics, but the ΔAIC revealed significant differences among model fits among the three morphometric variables. Based on the overall weight of evidence, eye diameter provided a more comprehensive depiction of the change in the reliance on copepod nauplii, along with greater predictive capability than the other metrics. Foraging prey–predator interactions by larval fish consist of a sequence of processes: search, encounter, recognition, reaction, attack and capture (handling and ingestion). Eye diameter, as an index of visual acuity (Blaxter, 1968, 1986; Poling & Fuiman, 1997), is the only metric which contributed to most or all of these processes, while body length had stronger influences on search (Leis, 2007; Peck et al., 2012), attack and capture ability (Heath, 1993; Morley & Batty, 1996), and maxilla length had direct effects on whether a prey can be ingested (Munk & Kiørboe, 1985; Pepin & Penney, 1997; Shirota, 1970). Eye development was also linked to aspects of the cognitive and behavioural responses of larvae to prey (Browman & O'Brien, 1992; Bruno et al., 2018; Munk, 1995; Vollset et al., 2011). The greater precision with which eye diameter can be measured and the greater differentiation in the rate of developmental change among fish species, relative to maxilla length, may contribute to the value of this morphological metric in explaining the transition from feeding on nauplii to copepodites in the diet. There also remains considerable uncertainty in the general logistic model that is probably affected by factors related to capture ability and prey behavioural responsiveness, which may explain the relationship of residuals to body length. The multitude of ways in which eye development may contribute to foraging capacity may provide some rationale about the value of this metric in providing a reliable foundational model to describe the shift from nauplii to copepodites in marine fish larvae. The general model and principles identified through the analyses from this study may serve to assess the range of possible outcomes of process‐oriented models and evaluate the uncertainty of their predictions (Daewel et al., 2008; Fiksen & MacKenzie, 2002; Hufnagl & Peck, 2011).

The challenge in understanding prey selection in larval fish, or in comparisons among species or ecosystems, lies in determining what level of detail is necessary to identify some general principles that can help define expectations through ontogenetic development. Peck et al. (2012) were able to identify phylogenetic commonalities in the patterns of covariance among body dimensions such as jaw length and eye diameter, and argued that such basic features in the morphology can help to characterize general patterns in prey selection. However, beyond frequently measured body metrics, other morphological features, such as development of fins (Fisher & Hogan, 2007), shape and length of the gut (Govoni et al., 1986), body and head shape (Ferron & Leggett, 1994; Peck et al., 2012), and the occurrence and development of other feeding structures (Deary, 2020), are also likely to contribute to phylogenetic differentiation in feeding capacity that would likely affect the transition from nauplii to copepodites in larval diets. Furthermore, behavioural adjustments relative to prey characteristics and aggregation will change during early ontogeny (Browman & O'Brien, 1992; Hunter, 1972; Munk & Kiørboe, 1985), which may not be effectively described from simple measures of development or body form. Additionally, differences in larval reaction to different prey types along with other behavioural characteristics (Peck et al., 2012; Pepin, 2023), as well as differences in zooplankton community structure and ecosystems (Heath & Lough, 2007; Llopiz, 2013; Pepin, 2024), are also likely to affect the transition between copepod developmental stages. Furthermore, differences in the responsiveness and escape ability of different nauplii and copepodite prey are likely to influence departures from a general relationship (Borg et al., 2012; Burdick et al., 2007; Hwang & Turner, 1995; Titelman, 2001), which may vary seasonally depending on the seasonal occurrence of fish larvae (Pepin, 2023). How the array of developmental features may affect the value of the general model reported in this study will have to be assessed for other species or ecosystems to determine whether they can explain departures from the expectations or provide better predictions of the change in diet during larval development.

There is growing evidence that the potential importance and role of ciliates and protists to feeding during the early larval stages may be underestimated from traditional approaches used in diet analysis (Denis et al., 2018; Montagnes et al., 2010; Yebra et al., 2019; Zingel et al., 2019). Furthermore, shifts in ecosystem trophodynamics may result in changes in regional and stock productivity over time (Swalethorp et al., 2023). As a result, ontogenetic shifts in the copepod stages represented by the general empirical models described in this study will not represent the potential contribution of microplankton in the diet of larval fish. Pepin and Dower (2007) noted that larval witch flounder and capelin feed significantly on phytoplankton and heterotrophic protists, respectively. However, that study also noted that most species in this ecosystem do not appear to rely heavily on these types of prey in their overall diet, and Pepin (2023) noted that protists were found in ~2% of larvae from this dataset. At this stage, however, it is not possible to assess how reliance on prey other than metazoan plankton, particularly in terms of the contribution of nauplii to the diet, would change the analyses presented here. More comprehensive observations about the dependence of fish larvae on elements of the microbial loop and how these compare to current knowledge about the importance of copepods to early life stages of fish are needed.

The pattern of prey selection by radiated shanny represents a significant outlier to the predictions of the general model derived from this study (Figures 2 and S5), with the least rapid transition from nauplii to copepodites among the 11 species of this study. Radiated shanny are robust larvae with a convoluted gut that likely limits concerns about possible regurgitation of stomach contents on capture by plankton nets (Dower et al., 1998). Young et al. (2010), contrasting several cohorts of radiated shanny, noted ontogenetic shifts from nauplii to copepodites in all studies but they identified important differences in the rates of change in mean prey size and niche breadth among the six cohorts. Radiated shanny from the current study (1985/86) had the lowest weight‐specific consumption rates (SPC), with the majority of individual larvae at the extreme lower end of observations from the other cohorts, as well as the lowest length‐dependent rate of increase in mean prey size, but the highest rate of increase in niche breadth. Although average prey abundance from plankton samples from this study was high or comparable to many other years from Young et al. (2010), they noted a particularly strong positive relationship between SPC and niche breadth in the specimens from this study, which may be indicative of some form of prey limitation. It is possible that differences and variability in prey patchiness (Lough & Broughton, 2007; Owen, 1989; Young et al., 2009) or prey community composition may have resulted in shanny from the 1985/1986 study feeding on whatever prey are encountered, resulting in a wider niche breath. This may have resulted in a slower transition in reliance on copepod nauplii relative to other fish taxa from the current study, which may have been less affected than radiated shanny by food limitation.

Prey perception is a critical determinant of feeding success of the early life stages of fish. Several studies have documented the ontogeny of visual structures, many of which appear to indicate that visual plasticity is largely a pre‐programmed developmental sequence of events (Higgs & Fuiman, 1996; Shand et al., 2008; Valen et al., 2018). The rapid changes in size, morphology and behaviour that occur from hatch to metamorphosis are invariably related to changes in visual acuity and capacity, which are in turn linked to the larva's habitat (Higgs & Fuiman, 1998; Osse, 1990; Pankhurst & Eagar, 1996; Peña et al., 2003; Taylor et al., 2015). The functional relationships identified in this study may guide the formulation of trophodynamic models of prey–predator during the early life stages, particularly when coupled with the dynamics of lower trophic levels (e.g. Akimova et al., 2016; Hufnagl & Peck, 2011). However, the high degree of covariation among larval attributes during ontogeny makes it difficult to determine which factors are the primary determinants of foraging ability, as was evident from the comparative analyses presented in this study. The results indicate that greater predictive capacity of the transition from reliance on nauplii to copepodites can be achieved from a generalized model based on eye diameter and consistent with the potential role of visual acuity during the foraging sequence. Improved comparative assessments of ontogenetic changes in foraging potential and sensitivity of the larval stages of fish may be achieved through a comprehensive synthesis of the developmental progression in the functional morphology of the retina, and the consequences for the ontogenetic shift in visual acuity and behavioural responsiveness to microplankton.

## AUTHOR CONTRIBUTIONS

The samples from this study were collected by R. Penney, formerly with Fisheries and Oceans Canada. Pierre Pepin conceptualized the goals, analyses and interpretation of the results, and is the sole author this study.

## CONFLICT OF INTEREST STATEMENT

The author has no conflicts of interest to declare.

## ETHICS STATEMENT

The current study was based on existing data, therefore no ethical approval was needed. At the time of sample collection, no guidelines or policies concerning the collection of plankton samples had been developed by the Canadian Council for Animal Care.

## Supporting information


**DATA S1** Supporting Information.

## Data Availability

The raw data from this study are available through the Canada.ca Open Government data repository, at https://open.canada.ca/data/en/dataset/c981aac9-d8e8-459d-88b3-8b2880845e2b.

## References

[jfb70014-bib-0001] Akimova, A. , Hufnagl, M. , Kreus, M. , & Peck, M. A. (2016). Modeling the effects of temperature on the survival and growth of North Sea cod (*Gadus morhua*) through the first year of life. Fisheries Oceanography, 25, 193–209. 10.1111/fog.12145

[jfb70014-bib-0002] Blaxter, J. H. S. (1968). Visual thresholds and spectral sensitivity of herring larvae. Journal of Experimental Biology, 48, 39–53.

[jfb70014-bib-0003] Blaxter, J. H. S. (1986). Development of sense‐organs and behavior of teleost larvae with special reference to feeding and predator avoidance. Transactions of the American Fisheries Society, 115, 98–114.

[jfb70014-bib-0004] Borg, C. M. A. , Bruno, E. , & Kiørboe, T. (2012). The kinematics of swimming and relocation jumps in copepod nauplii. PLoS One, 7(10), e47486. 10.1371/journal.pone.0047486 23115647 PMC3480368

[jfb70014-bib-0005] Browman, H. I. , & O'Brien, W. J. (1992). The ontogeny of search behaviour in the white crappie, *Pomoxis annulamis* . Environmental Biology of Fishes, 34, 181–195.

[jfb70014-bib-0006] Bruno, E. , Hojgaard, J. K. , Hansen, B. W. , Munk, P. , & Stottrup, J. G. (2018). Influence of swimming behavior of copepod nauplii on feeding of larval turbot (*Scophthalmus maximus*). Aquaculture International, 26, 225–236. 10.1007/s10499-017-0199-x

[jfb70014-bib-0007] Burdick, D. S. , Hartline, D. K. , & Lenz, P. H. (2007). Escape strategies in co‐occurring calanoid copepods. Limnology and Oceanography, 52, 2373–2385. 10.4319/lo.2007.52.6.2373

[jfb70014-bib-0008] Burnham, K. P. , & Anderson, D. R. (2002). Model selection and multimodel inference: A practical information‐theoretic approach (2nd ed., p. 488). Springer‐Verlag.

[jfb70014-bib-0009] Daewel, U. , Peck, M. A. , Kuhn, W. , St John, M. A. , Alekseeva, I. , & Schrum, C. (2008). Coupling ecosystem and individual‐based models to simulate the influence of environmental variability on potential growth and survival of larval sprat (*Sprattus sprattus* L.) in the North Sea. Fisheries Oceanography, 17, 333–351. 10.1111/j.1365-2419.2008.00482.x

[jfb70014-bib-0010] Deary, A. L. (2020). Influence of feeding structures and early development on foraging guild assignment in four co‐occurring fishes (family Sciaenidae). Marine Biology, 167, 51. 10.1007/s00227-020-3661-7

[jfb70014-bib-0011] Deary, A. L. , Latour, R. J. , & Hilton, E. J. (2017). Niche partitioning in early life history stage, estuarine‐dependent fishes (Sciaenidae). Estuaries and Coasts, 40, 1757–1770. 10.1007/s12237-017-0248-8

[jfb70014-bib-0012] Denis, J. , Vincent, D. , Antajan, E. , Vallet, C. , Mestre, J. , Lefebvre, V. , Caboche, J. , Cordier, R. , Marchal, P. , & Loots, C. (2018). Gut fluorescence technique to quantify pigment feeding in downs herring larvae. Marine Ecology Progress Series, 607, 129–142. 10.3354/meps12775

[jfb70014-bib-0013] Dower, J. F. , Pepin, P. , & Leggett, W. C. (1998). Enhanced gut fullness and an apparent shift in size selectivity by radiated shanny (*Ulvaria subbifurcata*) larvae in response to increased turbulence. Canadian Journal of Fisheries and Aquatic Sciences, 55, 128–142.

[jfb70014-bib-0014] Ferron, A. , & Leggett, W. C. (1994). An appraisal of body condition measures for marine fish larvae. Advances in Marine Biology, 30, 217–303.

[jfb70014-bib-0015] Fiksen, Ø. , & MacKenzie, B. R. (2002). Process‐based models of feeding and prey selection in larval fish. Marine Ecology Progress Series, 243, 151–164. 10.3354/meps243151

[jfb70014-bib-0016] Fisher, R. , & Hogan, J. D. (2007). Morphological predictors of swimming speed: A case study of pre‐settlement juvenile coral reef fishes. Journal of Experimental Biology, 210, 2436–2443. 10.1242/jeb.004275 17601947

[jfb70014-bib-0017] Govoni, J. J. , Boehlert, G. W. , & Watanabe, Y. (1986). The physiology of digestion in fish larvae. Environmental Biology of Fishes, 16, 59–77. 10.1007/bf00005160

[jfb70014-bib-0018] Heath, M. R. (1993). The role of escape reactions in determining the size distribution of prey captured by herring larvae. Environmental Biology of Fishes, 38, 331–344. 10.1007/bf00007527

[jfb70014-bib-0019] Heath, M. R. , & Lough, R. G. (2007). A synthesis of large‐scale patterns in the planktonic prey of larval and juvenile cod (*Gadus morhua*). Fisheries Oceanography, 16, 169–185. 10.1111/j.1365-2419.2006.00423.x

[jfb70014-bib-0020] Higgs, D. M. , & Fuiman, L. A. (1996). Ontogeny of visual and mechanosensory structure and function in Atlantic menhaden *Brevoortia tyrannus* . Journal of Experimental Biology, 199, 2619–2629. 9320556 10.1242/jeb.199.12.2619

[jfb70014-bib-0021] Higgs, D. M. , & Fuiman, L. A. (1998). Associations between behavioural ontogeny and habitat change in clupeoid larvae. Journal of the Marine Biological Association of the United Kingdom, 78, 1281–1294. 10.1017/s0025315400044490

[jfb70014-bib-0022] Hjort, J. (1914). Fluctuations in the great fisheries of northern Europe viewed in the light of biological research. Rapport Des Procès Verbaux Des Réunions du Conseil International Pour l'Exploration de la Mer, 20, 1–228.

[jfb70014-bib-0023] Houde, E. D. (2008). Emerging from Hjort's shadow. Journal of the Northwest Atlantic Fisheries Organization, 41, 53–70.

[jfb70014-bib-0024] Hufnagl, M. , & Peck, M. A. (2011). Physiological individual‐based modelling of larval Atlantic herring (*Clupea harengus*) foraging and growth: Insights on climate‐driven life‐history scheduling. ICES Journal of Marine Science, 68, 1170–1188. 10.1093/icesjms/fsr078

[jfb70014-bib-0025] Hunter, J. R. (1972). Swimming and feeding behavior of larval anchovy *Engraulis mordax* . Fishery Bulletin of the National Oceanic and Atmospheric Administration, 70, 821–838.

[jfb70014-bib-0026] Hunter, J. R. (1981). Feeding ecology and predation of marine fish larvae. In R. Lasker (Ed.), Marine fish larvae: Morphology, ecology and relation to fisheries (pp. 33–79). Washington Sea Grant Program.

[jfb70014-bib-0027] Hwang, J. S. , & Turner, J. T. (1995). Behaviour of cyclopoid, harpacticoid, and calanoid copepods from coastal waters of Taiwan. Marine Ecology‐Pubblicazioni Della Stazione Zoologica Di Napoli I, 16, 207–216. 10.1111/j.1439-0485.1995.tb00406.x

[jfb70014-bib-0028] Last, J. M. (1978a). The food of four species of pleuronectiform larvae in the eastern English Channel and southern North Sea. Marine Biology, 45, 359–368.

[jfb70014-bib-0029] Last, J. M. (1978b). The food of three species of gadoid larvae in the eastern English Channel and southern North Sea. Marine Biology, 48, 377–386.

[jfb70014-bib-0030] Leis, J. M. (2007). Behaviour as input for modelling dispersal of fish larvae: Behaviour, biogeography, hydrodynamics, ontogeny, physiology and phylogeny meet hydrography. Marine Ecology Progress Series, 347, 185–193.

[jfb70014-bib-0031] Llopiz, J. K. (2013). Latitudinal and taxonomic patterns in the feeding ecologies of fish larvae: A literature synthesis. Journal of Marine Systems, 109, 69–77. 10.1016/j.jmarsys.2012.05.002

[jfb70014-bib-0032] Lough, R. G. , & Broughton, E. A. (2007). Development of micro‐scale frequency distributions of plankton for inclusion in foraging models of larval fish, results from a video plankton recorder. Journal of Plankton Research, 29, 7–17. 10.1093/plankt/fb1055

[jfb70014-bib-0033] Maillet, G. , Belanger, D. , Doyle, G. , Robar, A. , Rastin, S. , Ramsay, D. , & Pepin, P. (2022). Optical, chemical and biological oceanographic conditions on the Newfoundland and Labrador shelf during 2018. Canadian Science Advisory Secretariat Research Document, viii–53.

[jfb70014-bib-0034] Miller, T. J. , Crowder, L. B. , Rice, J. A. , & Binkowski, F. P. (1992). Body size and the ontogeny of the functional‐response in fishes. Canadian Journal of Fisheries and Aquatic Sciences, 49, 805–812. 10.1139/f92-091

[jfb70014-bib-0035] Miller, T. J. , Crowder, L. B. , Rice, J. A. , & Marschall, E. A. (1988). Larval size and recruitment mechanisms in fishes – toward a conceptual framework. Canadian Journal of Fisheries and Aquatic Sciences, 45, 1657–1670. 10.1139/f88-197

[jfb70014-bib-0036] Montagnes, D. J. S. , Dower, J. F. , & Figueiredo, G. M. (2010). The protozooplankton‐ichthyoplankton trophic link: An overlooked aspect of aquatic food webs. Journal of Eukaryotic Microbiology, 57, 223–228. 10.1111/j.1550-7408.2010.00476.x 20384906

[jfb70014-bib-0037] Morley, S. A. , & Batty, R. S. (1996). The effects of temperature on “S‐strike” feeding of larval herring, *Clupea harengus* L. Marine and Freshwater Behaviour and Physiology, 28, 123–136. 10.1080/10236249609378983

[jfb70014-bib-0038] Morote, E. , Olivar, M. P. , Villate, F. , & Uriarte, I. (2010). A comparison of anchovy (*Engraulis encrasicolus*) and sardine (*Sardina pilchardus*) larvae feeding in the Northwest Mediterranean: Influence of prey availability and ontogeny. ICES Journal of Marine Science, 67, 897–908. 10.1093/icesjms/fsp302

[jfb70014-bib-0039] Morote, E. , Olivarl, M. P. , Pankhurst, P. M. , Villate, F. , & Uriarte, M. (2008). Trophic ecology of bullet tuna *Auxis rochei* larvae and ontogeny of feeding‐related organs. Marine Ecology Progress Series, 353, 243–254. 10.3354/meps07206

[jfb70014-bib-0040] Munk, P. (1992). Forgaing behavior and prey size spectra of larval herring *Clupea harengus* . Marine Ecology Progress Series, 80, 149–158. 10.3354/meps080149

[jfb70014-bib-0041] Munk, P. (1995). Foraging behaviour of larval cod (*Gadus morhua*) influenced by prey density and hunger. Marine Biology, 122, 205–212.

[jfb70014-bib-0042] Munk, P. , & Kiørboe, T. (1985). Feeding behavior and swimming activity of larval herring (*Clupea harengus*) in relation to density of copepod nauplii. Marine Ecology Progress Series, 24, 15–21. 10.3354/meps024015

[jfb70014-bib-0043] Nash, R. D. M. , Valencia, A. H. , & Geffen, A. J. (2006). The origin of Fulton's condition factor ‐ setting the record straight. Fisheries, 31, 236–238.

[jfb70014-bib-0044] Nunn, A. D. , Tewson, L. H. , & Cowx, I. G. (2012). The foraging ecology of larval and juvenile fishes. Reviews in Fish Biology and Fisheries, 22, 377–408. 10.1007/s11160-011-9240-8 26935792

[jfb70014-bib-0045] Osse, J. W. M. (1990). Form changes in fish larvae in relation to changing demands of function. Netherlands Journal of Zoology, 40, 362–385. 10.1163/156854289x00354

[jfb70014-bib-0046] Owen, R. W. (1989). Mcroscale and fine‐scale variations of small plankton in coastal and pelagic environments. Journal of Marine Research, 47, 197–240.

[jfb70014-bib-0047] Pankhurst, P. M. , & Eagar, R. (1996). Changes in visual morphology through life history stages of the New Zealand snapper, *Pagrus auratus* . New Zealand Journal of Marine and Freshwater Research, 30, 79–90. 10.1080/00288330.1996.9516698

[jfb70014-bib-0048] Peck, M. A. , Huebert, K. B. , & Llopiz, J. K. (2012). Intrinsic and extrinsic factors driving match‐mismatch dynamics during the early life history of marine dishes. In G. Woodward , U. Jacob , & E. J. Ogorman (Eds.), Advances in Ecological Research, 47: Global Change in Multispecies Systems, Pt 2 (pp. 177–302). Elsevier Ltd. 10.1016/b978-0-12-398315-2.00003-x

[jfb70014-bib-0049] Peña, R. , Dumas, S. , Villalejo‐Fuerte, M. , & Ortíz‐Galindo, J. L. (2003). Ontogenetic development of the digestive tract in reared spotted sand bass *Paralabrax maculatofasciatus* larvae. Aquaculture, 219, 633–644. 10.1016/s0044-8486(02)00352-6

[jfb70014-bib-0050] Pepin, P. (1995). An analysis of the length‐weight relation of larval fish – limitations of the general allometric model. Fishery Bulletin, 93, 419–426.

[jfb70014-bib-0051] Pepin, P. (2023). Feeding by larval fish: How taxonomy, body length, mouth size, and behaviour contribute to differences among individuals and species from a coastal ecosystem. ICES Journal of Marine Science, 80, 91–106. 10.1093/icesjms/fsac215

[jfb70014-bib-0052] Pepin, P. (2024). Foraging by larval fish: A full stomach is indicative of high performance but random encounters with prey are also important. ICES Journal of Marine Science, 81(4), 790–806. 10.1093/icesjms/fsae037

[jfb70014-bib-0053] Pepin, P. , Colbourne, E. , & Maillet, G. (2011). Seasonal patterns in zooplankton community structure on the Newfoundland and Labrador shelf. Progress in Oceanography, 91, 273–285. 10.1016/j.pocean.2011.01.003

[jfb70014-bib-0054] Pepin, P. , & Dower, J. E. (2007). Variability in the trophic position of larval fish in a coastal pelagic ecosystem based on stable isotope analysis. Journal of Plankton Research, 29, 727–737. 10.1093/plankt/fbm052

[jfb70014-bib-0055] Pepin, P. , Dower, J. F. , & Leggett, W. C. (1998). Changes in the probability density function of larval fish body length following preservation. Fishery Bulletin, 96, 633–640.

[jfb70014-bib-0056] Pepin, P. , & Penney, R. W. (1997). Patterns of prey size and taxonomic composition in larval fish: Are there general size‐dependent models? Journal of Fish Biology, 51, 84–100.

[jfb70014-bib-0057] Poling, K. R. , & Fuiman, L. A. (1997). Sensory development and concurrent behavioural changes in Atlantic croaker larvae. Journal of Fish Biology, 51, 402–421.

[jfb70014-bib-0058] Ricker, W. E. (1975). Computation and interpretation of biological statistics of fish populations (Vol. 191, pp. 1–382). Bulletin of the Fisheries Research Board of Canada.

[jfb70014-bib-0059] Ronnestad, I. , Yufera, M. , Ueberschar, B. , Ribeiro, L. , Saele, O. , & Boglione, C. (2013). Feeding behaviour and digestive physiology in larval fish: Current knowledge, and gaps and bottlenecks in research. Reviews in Aquaculture, 5, S59–S98. 10.1111/raq.12010

[jfb70014-bib-0060] Shand, J. , Davies, W. L. , Thomas, N. , Balmer, L. , Cowing, J. A. , Pointer, M. , Carvalho, L. S. , Trezise, A. E. O. , Collin, S. P. , Beazley, L. D. , & Hunt, D. M. (2008). The influence of ontogeny and light environment on the expression of visual pigment opsins in the retina of the black bream, *Acanthopagrus butcheri* . Journal of Experimental Biology, 211, 1495–1503. 10.1242/jeb.012047 18424684

[jfb70014-bib-0061] Shirota, A. (1970). Studies on the mouth size of fish larvae. Nippon Suisan Gakkaishi (Bulletin of the Japanese Society of Scientific Fisheries), 36, 353–368. https://waves-vagues.dfo-mpo.gc.ca/Library/28493.pdf, 10.2331/suisan.36.353

[jfb70014-bib-0062] Swalethorp, R. , Landry, M. R. , Semmens, B. X. , Ohman, M. D. , Aluwihare, L. , Chargualaf, D. , & Thompson, A. R. (2023). Anchovy boom and bust linked to trophic shifts in larval diet. Nature Communications, 14, 7412. 10.1038/s41467-023-42966-0 PMC10698165 38052790

[jfb70014-bib-0063] Taylor, S. M. , Loew, E. R. , & Grace, M. S. (2015). Ontogenic retinal changes in three ecologically distinct elopomorph fishes (Elopomorpha: Teleostei) correlate with light environment and behavior. Visual Neuroscience, 32, E005. 10.1017/s0952523815000024 26241034

[jfb70014-bib-0064] Titelman, J. (2001). Swimming and escape behavior of copepod nauplii: Implications for predator‐prey interactions among copepods. Marine Ecology Progress Series, 213, 203–213. 10.3354/meps213203

[jfb70014-bib-0065] Valen, R. , Karlsen, R. , & Helvik, J. V. (2018). Environmental, population and life‐stage plasticity in the visual system of Atlantic cod. Journal of Experimental Biology, 221(1), jeb165191. 10.1242/jeb.165191 29146770

[jfb70014-bib-0066] Vollset, K. W. , Folkvord, A. , & Browman, H. I. (2011). Foraging behaviour of larval cod (*Gadus morhua*) at low light intensities. Marine Biology, 158, 1125–1133. 10.1007/s00227-011-1635-5 24391266 PMC3873023

[jfb70014-bib-0067] von Herbing, I. H. , & Gallager, S. M. (2000). Foraging behavior in early Atlantic cod larvae (*Gadus morhua*) feeding on a protozoan (*Balanion* sp.) and a copepod nauplius (*Pseudodiaptomus* sp.). Marine Biology, 136, 591–602. 10.1007/s002270050719

[jfb70014-bib-0068] Williams, P. J. , Brown, J. A. , Gotceitas, V. , & Pepin, P. (1996). Developmental changes in escape response performance of five species of marine larval fish. Canadian Journal of Fisheries and Aquatic Sciences, 53, 1246–1253.

[jfb70014-bib-0069] Yebra, L. , de Rojas, A. H. , Valcárcel‐Pérez, N. , Castro, M. C. , García‐Gómez, C. , Cortés, D. , Mercado, J. M. , et al. (2019). Molecular identification of the diet of *Sardina pilchardus* larvae in the SW Mediterranean Sea. Marine Ecology Progress Series, 617, 41–52. 10.3354/meps12833

[jfb70014-bib-0070] Young, K. V. , Dower, J. F. , & Pepin, P. (2009). A hierarchical analysis of the spatial distribution of larval fish prey. Journal of Plankton Research, 31, 687–700. 10.1093/plankt/fbp017

[jfb70014-bib-0071] Young, K. V. , Pepin, P. , & Dower, J. F. (2010). Interannual variability in feeding rate and niche breadth of radiated shanny (*Ulvaria subbifurcata*) larvae from coastal Newfoundland. Journal of Plankton Research, 32, 815–827. 10.1093/plankt/fbq007

[jfb70014-bib-0072] Zingel, P. , Agasild, H. , Karus, K. , Buholce, L. , & Noges, T. (2019). Importance of ciliates as food for fish larvae in a shallow sea bay and a large shallow lake. European Journal of Protistology, 67, 59–70. 10.1016/j.ejop.2018.10.004 30453233

