# Supplementary material for: Morphological covariates of the ontogenetic shift from nauplii to copepodite prey in larval fish
Source: J Fish Biol. 2025 Mar 12;107(1):169–79. doi: 10.1111/jfb.70014 (PMC12327172; doi:10.1111/jfb.70014)
Supplement: Supplementary file 1 — DATA S1 Supporting Information. [file JFB-107-169-s001.docx]

# Supplemental material

# Morphological covariates of the ontogenetic shift from nauplii to copepodite prey in larval fish

Pierre Pepin^[[1]](#footnote-1)^

Northwest Atlantic Fisheries Centre, Fisheries and Oceans Canada, St. John’s, NL, Canada

DOI: <https://doi.org/10.1111/JFB.70014>

**Supplemental Table S1.** Body width of key copepod nauplii taxa from the diet of larval fish. Numbers represent the total occurrences among the stomach contents of the 11 species of fish included in the analyses. Illustrations of how measurements were taken are provided.

| Prey taxa | Stage | Width (µm) | Number | Illustrations |
| --- | --- | --- | --- | --- |
| *Calanus finmarchicus* | N I | 110 | 115 | 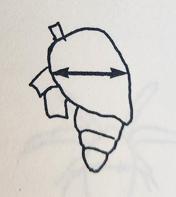 |
|  | N II | 135 | 292 |  |
|  | N III | 200 | 285 |  |
|  | N IV | 240 | 155 |  |
|  | N V | 275 | 45 |  |
|  | N VI | 305 | 19 |  |
| Calanoid nauplii | N I | 70 | 419 |  |
|  | N II | 90 | 1181 |  |
|  | N III | 120 | 950 |  |
|  | N IV | 150 | 428 |  |
|  | N V | 170 | 37 |  |
|  | N VI | 200 | 8 |  |
| *Temora longicornis* | N I | 55 | 25 | 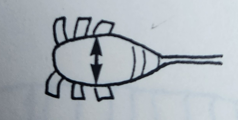 |
|  | N II | 80 | 142 |  |
|  | N III | 105 | 262 |  |
|  | N IV | 130 | 464 |  |
|  | N V | 160 | 378 |  |
|  | N VI | 190 | 95 |  |
| *Oithona similis* | N I | 50 | 21 | 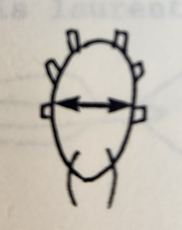 |
|  | N II | 65 | 51 |  |
|  | N III | 70 | 221 |  |
|  | N IV | 80 | 743 |  |
|  | N V | 95 | 743 |  |
|  | N VI | 105 | 215 |  |

**Supplemental Table S2**. Species specific intercept and slope of the linear relationship between larval maxilla (mm) and eye diameter (mm) versus body length (mm), and standard deviation (s.d.) of the residuals (mm), from Pepin (2024).

| Species | Length of maxilla versus larval length | | |  | Eye diameter versus larval length | | |
| --- | --- | --- | --- | --- | --- | --- | --- |
|  | Intercept | Slope | Residual s.d. |  | Intercept | Slope | Residual s.d. |
| *Clupea harengus* | -0.12 | 0.061 | 0.055 |  | -0.089 | 0.038 | 0.069 |
| *Mallotus villosus* | -0.009 | 0.046 | 0.048 |  | 0.051 | 0.028 | 0.057 |
| *Gadus morhua* | 0.009 | 0.103 | 0.095 |  | 0.054 | 0.070 | 0.054 |
| *Glyptocephalus cynoglossus* | -0.14 | 0.077 | 0.070 |  | 0.177 | 0.027 | 0.077 |
| *Myzopsetta ferruginea* | -0.025 | 0.086 | 0.064 |  | -0.014 | 0.065 | 0.035 |
| *Hippoglossoides platessoides* | -0.19 | 0.095 | 0.067 |  | 0.056 | 0.043 | 0.069 |
| *Pseudopleuronectes americanus* | 0.018 | 0.077 | 0.047 |  | 0.073 | 0.055 | 0.031 |
| *Tautogolabrus adspersus* | -0.056 | 0.096 | 0.060 |  | -0.088 | 0.095 | 0.062 |
| *Ulvaria subbifurcata* | 0.02 | 0.076 | 0.062 |  | 0.184 | 0.036 | 0.041 |
| *Stichaeus punctatus* | 0.001 | 0.072 | 0.070 |  | -0.154 | 0.061 | 0.076 |
| *Liparis* spp. | -0.15 | 0.084 | 0.078 |  | 0.166 | 0.039 | 0.023 |

**Supplemental Figure S1**. Regression lines of the relationships between eye diameter and body length (top panel), maxilla length and body length (middle panel), and derived relationship between maxilla length and eye diameter (bottom panel), for 11 species of larval fish.





**Supplemental Figure S2**. Functional relationships between eye diameter and body weight (top panel), maxilla length and body weight (middle panel), and body length and body weight (lower panel), derived from the relationships reported by Pepin (1995, 2024).


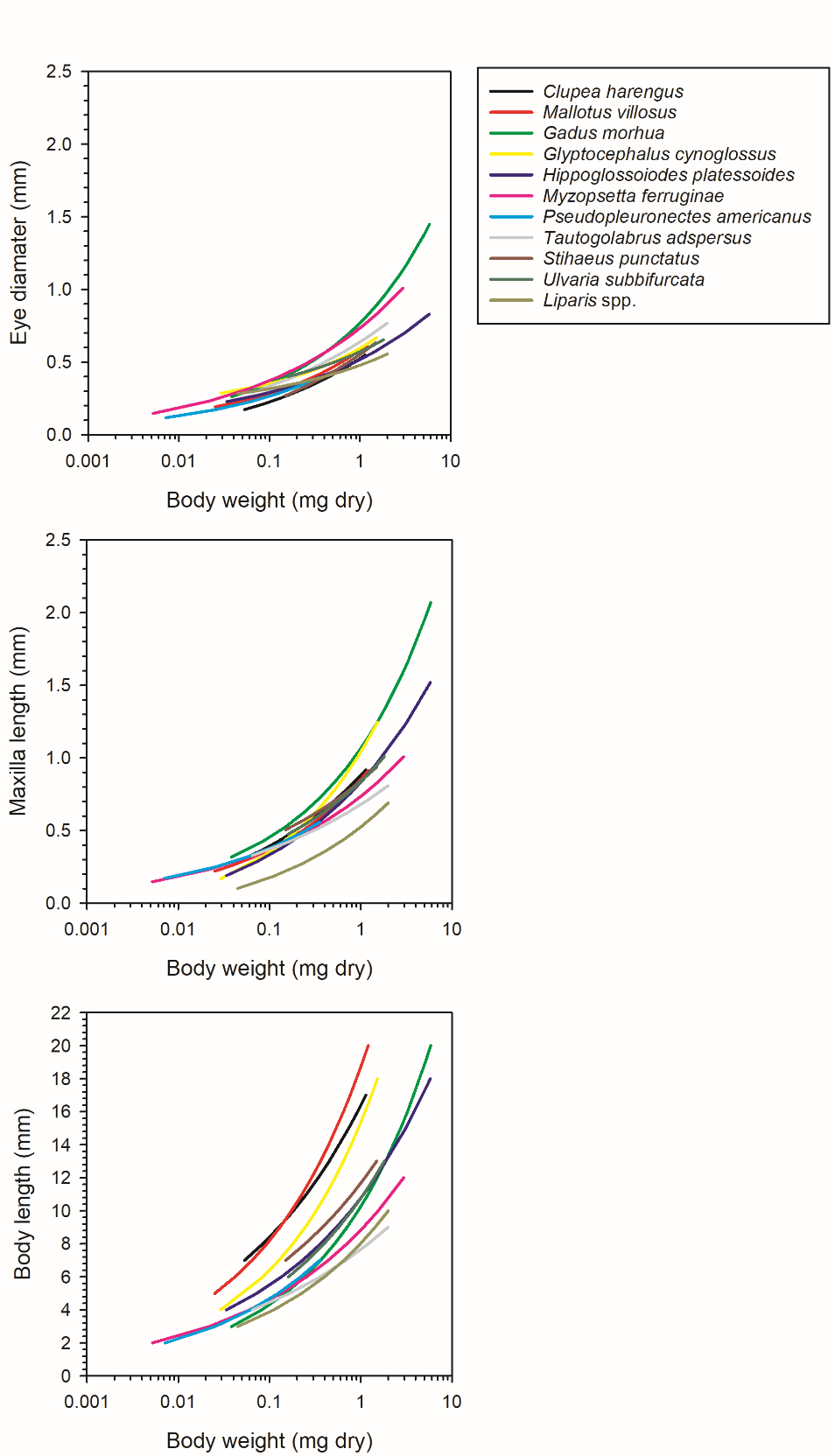


**Supplemental Figure S3**. Width of the largest nauplii in the stomach of each larval fish in relation to maxilla length. Gape is estimated as $\sqrt{2}\cdot Maxilla length$, assuming an opened mouth jaw angle of 90° (Shirota, 1970).





**Supplemental Figure S4**. Relative frequency distribution of nauplii (top panel) and copepodite (bottom panel) widths relative to gape ($\sqrt{2}\cdot Maxilla length$) for all larvae from this study, weighted by the overall occurrence in each interval of relative widths, and based on measurements of 7294 nauplii and 5535 copepodites from the stomachs of 11 fish species. Each bar represents intervals of 0.01 in relative widths. Blue reference lines represents the median for nauplii (0.180) and copepodites (0.205).


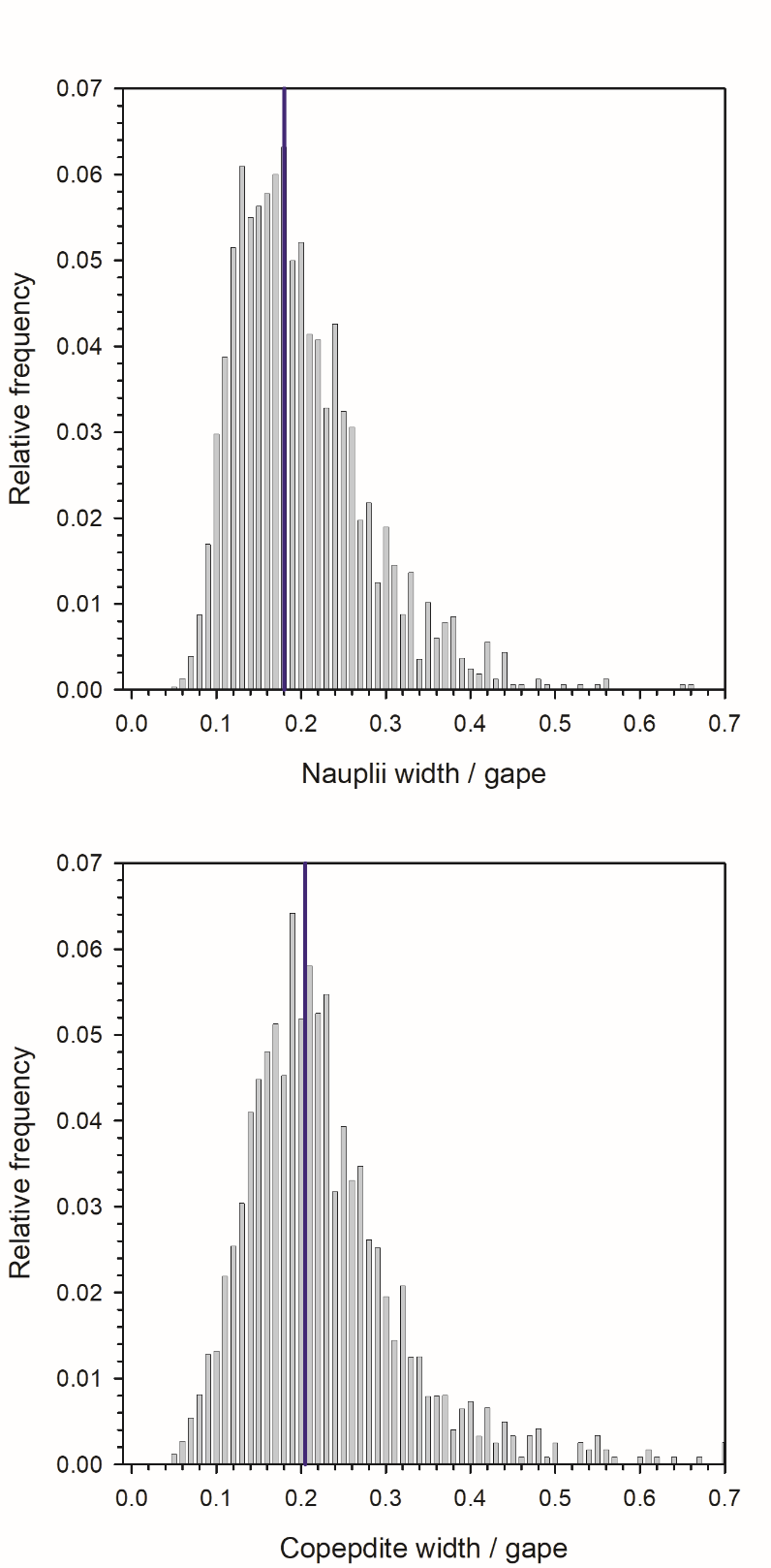


**Supplemental Figure S5**. Distribution of *Bonferroni* corrected significant differences from pairwise comparisons among fish species based on an analysis of variance of the residuals from logistic model fits for the proportion of copepod nauplii in relation to eye diameter (E – yellow), maxilla length (M – blue), and body length (L – orange). Filled squares represent significant differences at *P* ≤ 0.0009. Species-specific significant correlations (*P* < 0.05) of the residuals for the three logistic models with length and Fulton’s K are indicated by +/- signs for positive and negative relationships, respectively. Fulton’s condition index for each fish species was estimated based on the allometric relationships reported by Pepin (1995) ($K=1000*(W/L^{3})$; where *W* is weight in mg dry and *L* is length in mm (modified from Ricker, 1975) – see Nash et al. (2006); a greater *K* indicates increased body depth (i.e., mass) at a given length).


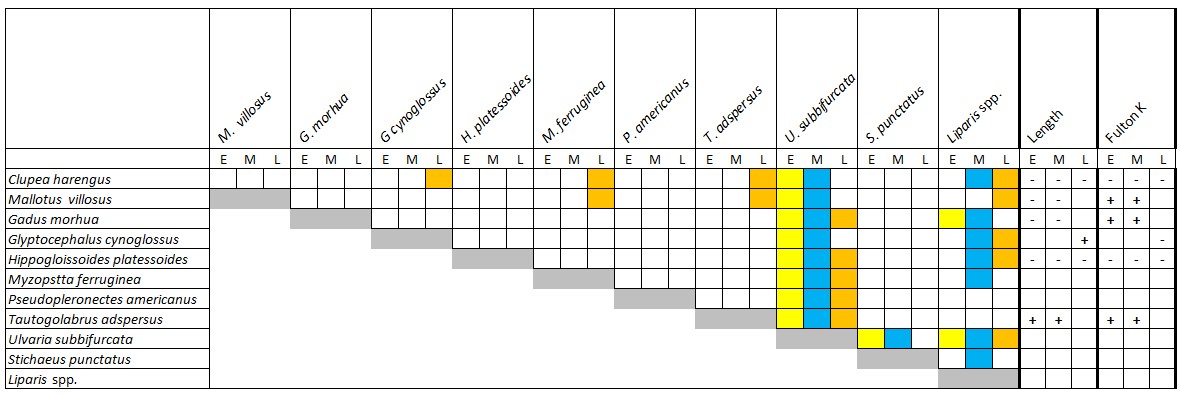


## References

Nash, R. D. M., Valencia, A. H., and Geffen, A. J. 2006. The origin of Fulton's condition factor - Setting the record straight. Fisheries, 31: 236-238.

Pepin, P. 1995. An analysis of the length-weight relation of larval fish - limitations of the general allometric model. Fishery Bulletin, 93: 419-426.

Pepin, P. 2024. Foraging by larval fish: a full stomach is indicative of high performance but random encounters with prey are also important. ICES Journal of Marine Science [ICES J. Mar. Sci.]. <https://doi.org/10.1093/icesjms/fsae037>

Ricker, W. E. 1975. Computation and interpretation of biological statistics of fish populations. Bulletin of the Fisheries Research Board of Canada, 191: 1-382.

Shirota, A. 1970. Studies on the mouth size of fish larvae. Nippon Suisan Gakkaishi (Bulletin of the Japanese Society of Scientific Fisheries). Translation of the Fisheries Research Board of Canada No. 1978, 36: 353-368. <https://waves-vagues.dfo-mpo.gc.ca/Library/28493.pdf> <https://doi.org/10.2331/suisan.36.353>

1. Corresponding author: [pierre.pepin.nl@gmail.com](mailto:pierre.pepin.nl@gmail.com); ORCID <https://orcid.org/0000-0003-4555-4843> [↑](#footnote-ref-1)
